# Inhaled Xenon Washout as a Biomarker of Alzheimer’s Disease

**DOI:** 10.3390/diagnostics8020041

**Published:** 2018-06-06

**Authors:** Francis T. Hane, Tao Li, Jennifer-Anne Plata, Ayman Hassan, Karl Granberg, Mitchell S. Albert

**Affiliations:** 1 Department of Chemistry, Lakehead University, 955 Oliver Rd, Thunder Bay, ON P7B 5E1, Canada; lit@tbh.net (T.L.); plataj@tbh.net (J.-A.P.); albertmi@tbh.net (M.S.A.); 2 Thunder Bay Regional Health Research Institute, 980 Oliver Rd, Thunder Bay, ON P7B 5E1, Canada; 3 Thunder Bay Regional Health Sciences Centre, 980 Oliver Rd, Thunder Bay, ON P7B 5E1, Canada; hassana@tbh.net (A.H.); granberk@tbh.net (K.G.); 4 Northern Ontario School of Medicine, 955 Oliver Rd, Thunder Bay, ON P7B 5E1, Canada

**Keywords:** hyperpolarized gas MRI, xenon, gas retention, Alzheimer’s disease, wash out, vascular

## Abstract

Biomarkers have the potential to aid in the study of Alzheimer’s disease (AD); unfortunately, AD biomarker values often have a high degree of overlap between healthy and AD individuals. This study investigates the potential utility of a series of novel AD biomarkers, the sixty second ^129^Xe retention time, and the xenon washout parameter, based on the washout of hyperpolarized ^129^Xe from the brain of AD participants following inhalation. The xenon washout parameter is influenced by cerebral perfusion, T1 relaxation of xenon, and the xenon partition coefficient, all factors influenced by AD. Participants with AD (*n* = 4) and healthy volunteers (*n* = 4) were imaged using hyperpolarized ^129^Xe magnetic resonance imaging (MRI) and magnetic resonance spectroscopy (MRS) to determine the amount of retained xenon in the brain. At 60 s after the breath hold, AD patients retained significantly higher amounts of ^129^Xe compared to healthy controls. Data was fit to a pharmacokinetic model and the xenon washout parameter was extracted. Xenon washout in white and grey matter occurs at a slower rate in Alzheimer’s participants (^129^Xe half-life time of 42 s and 43 s, respectively) relative to controls (20 s and 16 s, respectively). Following larger scale clinical trials for validation, the xenon washout parameter has the potential to become a useful biomarker for the support of AD diagnosis.

## 1. Introduction

Alzheimer’s disease (AD) is an age-related neurodegenerative disorder that compromises the memory and executive function of affected individuals [1]. The etiology of AD is complex, with a variety of factors such as genetics [2,3,4,5,6], the amyloid-β (Aβ) protein [7,8,9,10,11], the microtubule tau protein [8,12,13,14,15], and inflammation [16,17,18], amongst others, interacting with one another to influence the pathology and progression of AD. While the amyloid cascade hypothesis and its variants [7,19,20,21] remain the favored AD hypothesis, the exact cause of AD remains contentious [22,23,24,25].

Cerebro-vascular factors have been demonstrated to both influence and to be influenced by AD pathology [26,27,28]. These vascular effects include an increased risk of stroke in AD patients [29]. Paradoxically, a history of strokes excludes a diagnosis of AD in favor of vascular dementia [30]. This only muddies the waters in differentiating between these two causes of dementia and raises discordance: If an individual suffers a stroke and then presents with dementia symptoms, the dementia is likely to be differentiated as vascular dementia, yet if an AD patient suffers a stroke following diagnosis, the stroke is likely to be attributed to the AD pathology.

The various pathological factors involved in AD have allowed for the discovery of an abundance of AD biomarkers based on data obtained from medical imaging, cerebrospinal fluid and blood plasma [31,32,33,34,35,36]. Medical imaging, including positron emission tomography (PET) [37,38,39,40] and magnetic resonance imaging (MRI) [41,42,43,44,45], has provided a wealth of information on the pathology of AD both in research settings and as confirmatory diagnostic tools [38,46,47,48].

Impaired cerebral perfusion is one of the many pathological hallmarks of AD [49] and its use as a biomarker for AD was established even before the amyloid cascade hypothesis was first posited [50] in the early 1990s. Numerous research groups have observed a reduction in cerebral perfusion in AD patients using a variety of techniques [51,52,53]. In one such example, Perani, et al. showed a reduction in global cerebral perfusion compared to healthy controls using technetium-99m hexamethylpropyleneamine oxime ([99mTc]HM-PAO) Single Positron Emission Tomography (SPECT) [54]. Parkes and colleagues employed arterial spin labelling (ASL), an MRI-based technique, to demonstrate a decrease in the grey matter to white matter (GM:WM) perfusion ratio, which was attributed to the reduction in GM perfusion [55]. Work by Du et al. and Johnson et al. localized areas of hypoperfusion to the parietal cortex [56,57]. In addition to localizing impaired cerebral perfusion to the parietal cortices, Schuff, and colleagues localized impaired perfusion to the frontal cortices [56]. ASL and phase contrast MRI have been used to detect changes in cerebral blood flow (CBF) in order to stage AD disease severity [58,59].

In addition to detecting endogenous nuclei in the body (i.e., protons), MRI can also be used to detect exogeneous nuclei. A variety of techniques have been developed to increase the sensitivity of MRI to these exogeneous nuclei. One of these techniques involves hyperpolarizing the nuclei of a variety of elements such as ^3^He [60], ^129^Xe [61,62,63] and ^13^C [64], to align their nuclear spin angular momentum, providing an increase in the signal-to-noise (SNR) ratio of approximately five orders of magnitude [65]. Because these nuclei are not ubiquitous to the body under ordinary physiological conditions, they can be detected and tracked throughout the body using MRI and magnetic resonance spectroscopy (MRS) [66,67,68]. In particular, a hyperpolarized (HP) gas, ^129^Xe, dissolves into the blood following inhalation [69] and then travels throughout the vasculature and accumulates in highly perfused tissues such as the brain [70,71]. This process is referred to as xenon wash-in. Xenon then washes out of the brain tissue, dissolving in the blood and is then exhaled following gas exchange in the lungs.

The diagnosis of AD is a diagnosis of exclusion and is primarily based upon clinical presentation [30]. Biomarkers obtained from the cerebrospinal fluid (CSF) or from imaging may be used as confirmatory tools [72]. One of the difficulties in using AD-associated biomarkers for AD diagnosis is the large overlap in values between AD patients and healthy individuals; while the mean values between the two populations may be statistically significant, many AD patients may have biomarker values close to the “normal” range, and vice versa [73]. Abnormal Aβ42 levels are typically set at <550 ng/L [74]. This overlap has motivated the search for AD biomarkers with higher diagnostic accuracy. Recently, Nakamura, et al. demonstrated convincing plasma AD biomarkers at accuracies exceeding 90% using highly sensitive immunoprecipitation and mass spectrometry [75]. At present, biomarkers are ideally suited to quantitatively monitor disease progression over time, comparing current values to baseline values.

In the present study, we employed HP ^129^Xe, MRI and MRS to probe the xenon gas exchange characteristics in the brain of AD participants [63]. We demonstrate the preliminary results of two potential biomarkers of AD based on the washout of HP ^129^Xe from the brain of AD participants, which was detected using ^129^Xe MRS. The washout of the ^129^Xe from the brain can be used as an indirect measure of brain perfusion. In contrast to the high degree of overlap of many existing AD biomarkers, our preliminary data demonstrate a five standard deviation difference of ^129^Xe-GM signal retention at 60 s following the breath hold and a nearly two standard deviation difference in the xenon washout parameter between AD participants and healthy controls. The xenon washout parameter is calculated by fitting the xenon signal curve to a pharmacokinetic equation (see Equation (1) in results). The difference in xenon retention values between AD participants and healthy controls makes these potential biomarkers candidates for larger scale future studies.

## 2. Materials and Methods

### 2.1. Ethical Approval and Consent to Participate

This research study was approved by the research ethics boards (REB) of Lakehead University (LU) and the Thunder Bay Regional Health Sciences Centre (TBRHSC) (Reference number RP-307) and was conducted in accordance with the Tri-Council Policy Statement-2 (TCPS-2). All participants consented to their data being used for publication.

### 2.2. Participant Recruitment

Four participants diagnosed with mild to moderate AD were recruited from the community for participation in this study. AD patients were diagnosed using clinical criteria by a qualified neurologist or gerontologist. Additionally, four age-matched healthy volunteers were recruited to serve as controls. Age-matched control participants were all cognitively normal. Informed consent was obtained from all human participants.

### 2.3. ^1^H Magnetic Resonance Imaging

Participants were placed into a dual tuned ^1^H/^129^Xe head coil (Clinical MR Solutions LLC, Brookfield, WI, USA) in a Philips Achieva 3T clinical MRI scanner. T2-weighted ^1^H MRI was acquired using a turbo-spin echo (TSE) sequence with the following parameters: FOV = 250 mm × 250 mm, matrix = 256 × 256, TR/TE = 3 s/80 ms, NSA = 5, FA = 90°.

### 2.4. ^129^Xe Magnetic Resonance Spectroscopy

Enriched ^129^Xe was polarized to ~35% using a Xemed xenon (Xemed LLC, Durham, NH, USA) gas polarizer and dispensed into a 500 mL Tedlar bag. The participants inhaled the Xe gas and held their breath for 20 s. Sixty dynamic spectra were acquired every 2 s beginning with Xe inhalation. Xe MRS parameters were as follows: 60 dynamic scans, bandwidth 32 kHz, sample number: 4096, TR/TE = 2 s/0.17 ms, FA = 10°. The signal was a single voxel encompassing the entirety of the brain region. We used a low flip angle to maintain polarization of the ^129^Xe gas throughout all dynamic scans. Both Xe-GM and Xe-WM peaks were plotted as a function of time. Signal intensity was calculated by measuring the peak divided by the standard deviation of the noise.

### 2.5. ^129^Xe Magnetic Resonance Imaging

Enriched ^129^Xe was polarized to ~35% as described above and dispensed into two 1 L Tedlar bags. Acquisition parameters were as follows: FOV = 250 mm × 250 mm, matrix = 32 × 32, TR/TE = 250 ms/0.84 ms, NSA = 1, FA = 12.5°, Bandwidth 150 Hz/pixel. Three dynamic scans were acquired at 10 s, 20 s and 30 s following inhalation.

### 2.6. ^129^Xe Image Processing

All images were processed using a custom Matlab script that converted the raw data in k-space into an MR image using a Fast Fourier Transform (FFT) algorithm. SNR maps were created by dividing each pixel by the standard deviation of the noise. Complete details on how the SNR maps were created are contained within the supplementary information.

### 2.7. Xenon Washout Parameter Maps Image Processing

The three dynamic ^129^Xe MRI were processed as described above. A custom Matlab script was used to calculate the xenon washout parameter of each pixel as described above to create a “xenon washout parameter map”. Xenon washout parameter maps from all individuals were averaged to create a mean xenon washout parameter map for all AD participants and healthy age-matched controls. A mask was created to remove noise from outside the brain region for image clarity. The xenon washout parameter maps were overlaid on T2W anatomical MRI using GIMP v.2.8 image processing software.

### 2.8. Statistical Analysis

Data for all participants was aggregated and the means and standard deviations were calculated. 16 data points for healthy controls and 13 data points for AD were used. (Each participant had xenon washout measured 3 or 4 times). A Welsh’s *t*-test (2-tail, unpaired) was conducted to establish statistical significance. For all comparisons, *p* < 0.01. However, due to the preliminary nature of this study and small sample size, *p* values were not stated.

## 3. Results

### 3.1. Magnetic Resonance Spectroscopy

**Figure 1:**
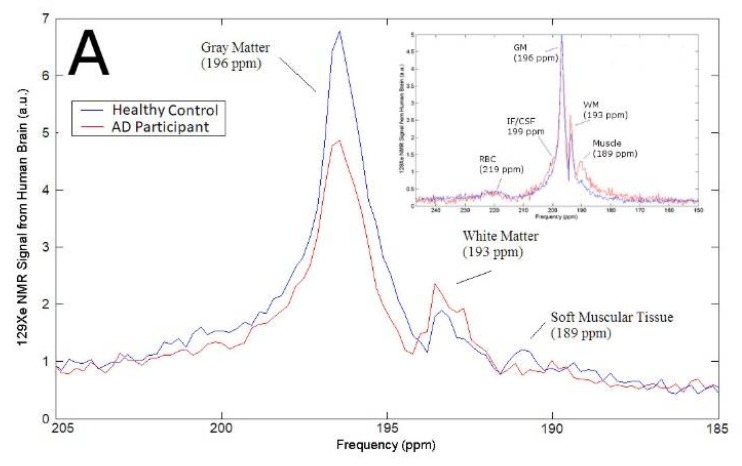

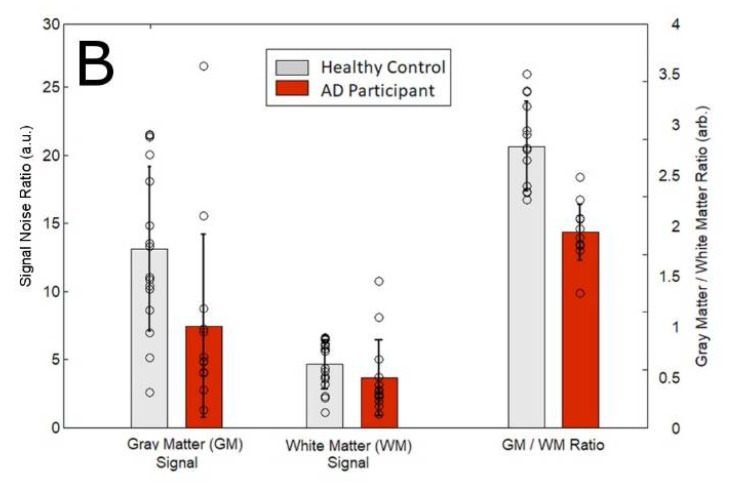
(**A**) Representative ^129^Xe MRS of healthy controls and AD participants. (**B**) Signal intensity of GM, WM, and the ratio of GM to WM in healthy controls compared to AD participants. a.u. = arbitrary units.

In this work, we analyzed the ^129^Xe magnetic resonance (MR) spectra from the brains of AD participants (*n* = 4) compared to healthy controls as a function of time. We began our study by ensuring that the ^129^Xe spectra we acquired were consistent with previously reported spectra. We observed five ^129^Xe MRS peaks measured at +189 ppm, +193 ppm, +196 ppm, +199 ppm, +219 ppm (Figure 1A). These peaks are consistent with previously reported spectra that assigned these peaks to ^129^Xe interacting with muscle, WM, GM, CSF and red blood cells (RBC), respectively [71,76]. The chemical shift of the ^129^Xe MRS acquired from AD participants did not differ appreciably from the MRS acquired from healthy controls. However, the signal intensity of brain matter peaks differed in AD participants versus healthy controls (Figure 1A). The ^129^Xe-GM signal was 43% lower in AD participants than in healthy controls. Whereas the ^129^Xe-WM was not statistically different between healthy controls and AD participants (Figure 1B). We also quantified the ratio of GM to WM. We calculated that the ^129^Xe-GM/^129^Xe-WM was 32% lower in AD participants than in healthy controls.

### 3.2. MRS as a Function of Time

**Figure 2:**
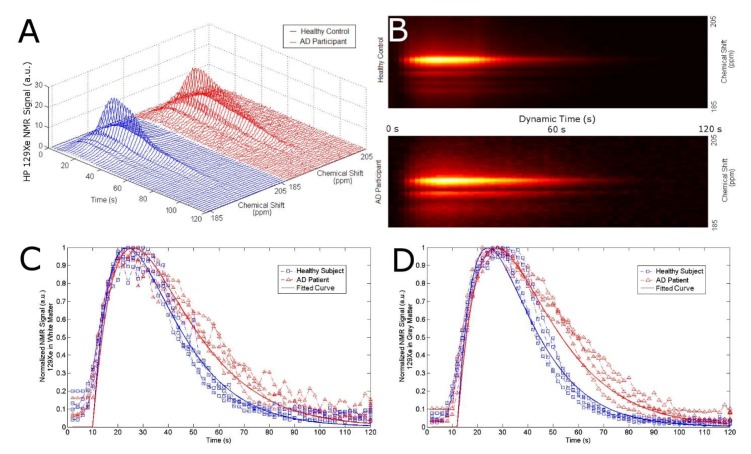
(**A**) Stack plot of dynamic ^129^Xe NMR spectra for healthy controls (blue) and AD patients (red). (**B**) Topographic “streak” plot of (**A**) depicting the NMR dynamic spectra from the top with SNR in “hotter” colors. Notice a higher SNR in AD patients for a longer time than that of the healthy controls. SNR of ^129^Xe-WM (**C**) and ^129^Xe-GM (**D**) as a function of time for healthy controls (blue) and AD participants (red). The participants inhaled 500 mL of HP ^129^Xe and held their breath for 20 s. ^129^Xe MRS from the brain region was acquired every 2 s. Notice an increase in ^129^Xe signal after approximately 10 s as the ^129^Xe reached the brain. At 20 s, the participant exhaled and the ^129^Xe signal began to decrease at different rates for AD participants vs. healthy controls for WM and GM.

**Figure 3:**
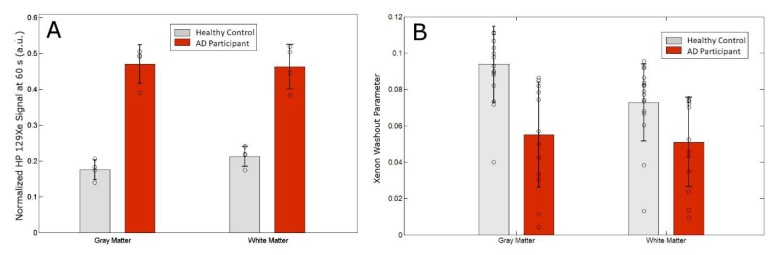
(**A**) Normalized ^129^Xe retention at 60 s after the breath-hold for Xe-WM and Xe-GM. (**B**) Xenon washout parameter for gray matter and white matter.

Next, we tracked the SNR of the ^129^Xe-WM and ^129^Xe-GM to probe the effect of AD on the washout time of ^129^Xe from the brain (Figure 2). We observed a considerable difference in ^129^Xe washout between AD participants and healthy controls. Healthy controls had a ^129^Xe washout half-life of 20 s and 16 s in WM and GM, respectively; while AD participants had a ^129^Xe washout half-life time of 42 s and 43 s in WM and GM, respectively. AD participants had a slower washout compared to healthy controls indicating increased ^129^Xe retention in the WM and GM of the brain. Sixty seconds (60 s) following ^129^Xe inhalation, we observed a five standard deviation difference in the ^129^Xe-GM between healthy controls and AD participants (Table 1, Figure 2D and Figure 3A). In fact, there was no overlap between healthy controls and AD participants.

We then quantified this washout time by applying the following pharmacokinetic model to the ^129^Xe washout signal [77]:

(1) S(t)=S(T)·e−β(t−T)·cos(t2−1)α

where β is the xenon washout parameter and is defined as,

(2) $$\beta =\;\frac{f}{p}+T1^{-1}$$

where S is the SNR as a function of time since exhalation, S(*T*) is the maximum xenon SNR at time *T*, *f* is the cerebral perfusion, *p* is the xenon partition coefficient, *T*1 is the ^129^Xe spin-lattice relaxation time in tissue, and α is the MR flip angle. By fitting the data to this model, we were able to extract the xenon washout parameter for each subject (Figure 3B).

We calculated a 41% and 31% reduction in the GM and WM xenon washout parameters, respectively, in AD patients compared to healthy controls (Table 1).

### 3.3. ^129^Xe MRI

**Figure 4:**
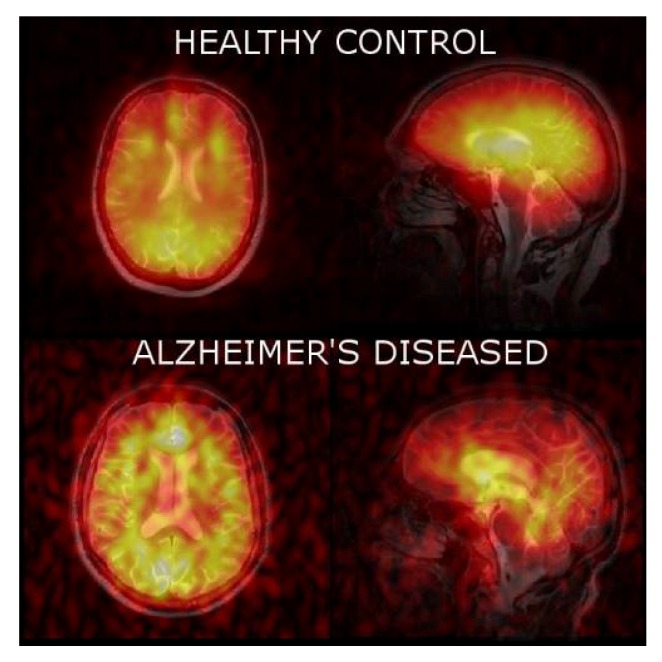
Axial and sagittal ^129^Xe MRI of healthy controls and AD participants. An observably higher SNR was obtained for healthy controls relative to AD participants.

Lastly, we acquired ^129^Xe MRI from the brain of healthy controls and AD participants (Figure 4). We were able to obtain significantly higher SNR images from healthy controls than from AD participants.

**Figure 5:**
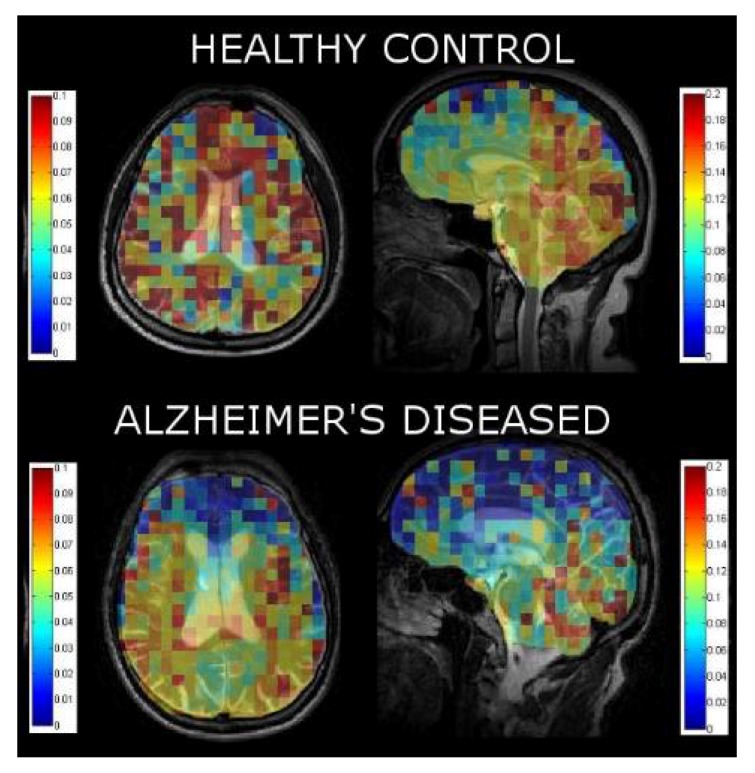
Xenon washout parameter maps of healthy controls age-matched to AD patients overlaid on T2W anatomical images.

By fitting the SNR of each voxel in the three dynamic images, we were able to calculate a xenon washout parameter in each pixel to create a xenon washout parameter map (Figure 5). Similar to our spectroscopic results, we qualitatively observe a higher xenon washout parameter in age-matched healthy controls (Figure 5, top) than in AD patients (Figure 5, bottom). Additionally, the xenon washout parameter remains similar in caudal brain regions in AD participants versus healthy controls, whereas there is a reduction in the localized xenon washout parameter in the prefrontal regions of AD participants.

## 4. Discussion

In this work, we made two significant findings. Firstly, AD participants have significantly lower ^129^Xe in the GM than healthy controls. Secondly, we found that AD subjects retain ^129^Xe within both the GM and WM of the brain significantly longer than healthy controls. Our results support the hypothesis that cerebral perfusion may be affected by AD pathology.

AD is considered primarily a disease of grey matter; however, white matter has been implicated as well in the AD pathology [78]. Our observed decrease in ^129^Xe-GM signal between AD participants and healthy controls suggests a reduction in GM volume or a decrease in ^129^Xe uptake in the GM. In contrast, the ^129^Xe-WM signal was not different between AD participants and healthy controls.

While there is considerable overlap in many AD biomarkers between healthy controls and AD patients, we calculated a five standard deviation difference in the ^129^Xe retention in the GM between AD patients compared to healthy controls (Table 1, Figure 2D and Figure 3A). Furthermore, no AD patients had ^129^Xe retention values below that of any healthy control and no healthy control had ^129^Xe retention values higher than the lowest AD ^129^Xe retention values.

Additionally, we introduce a potential AD biomarker that we denote as the xenon washout parameter from Equation (1). The measurement of xenon signal as a function of time (i.e., Xe washout) using ^129^Xe MRS was fit to a pharmacokinetic model (Equation (1)) and the xenon washout parameter was calculated. While other pharmacokinetic models [76] have been developed and fit our data, we utilized the model developed by Martin, et al. [77] because it was a better fit for our data as it is only applied to the washout phase and therefore relied on fewer assumptions than the model developed by Kilian et al. [76]. We calculated that the xenon washout parameter is nearly 2 standard deviations lower in AD participants than in healthy controls for grey matter.

In addition to our spectroscopic data, we were able to localize the xenon washout parameter to different brain regions. Our analysis of the localized xenon washout parameter from the xenon washout parameter maps indicates a lower xenon washout parameter in AD participants than in healthy controls (Figure 5). While this observation is consistent with our spectroscopic results, the xenon washout parameter obtained from imaging is less accurate than that obtained from spectroscopy because the model was fit using only three data points (three dynamic ^129^Xe images) for the imaging data compared to 60 data points for the spectroscopic data. From our qualitative observations of the sagittal β-parameter maps, we observed that the xenon washout parameter is higher in the caudal regions than it is in the frontal lobes (Figure 5) for both healthy controls and AD participants. In AD participants, while the mean xenon washout parameter decreases throughout the brain, the xenon washout parameter remains higher in the posterior regions of the brain. This observation could possibly indicate lower perfusion in the frontal lobes than in the caudal brain regions. This observation is consistent with previous reports indicating that AD pathology begins near the rostral regions of the brain and slowly migrates towards the caudal regions of the brain [79].

The cerebral perfusion, T1 of ^129^Xe in the brain, and the xenon partition coefficient all influence the xenon washout parameter as expressed in Equation (2). Cerebral perfusion has long been demonstrated to be reduced in AD patients [50,51,52,53,54,55,56,57,58]. Work by Binnewijzend et al. reported a difference of cerebral perfusion of approximately 27% which was one standard deviation lower in AD patients compared to healthy controls [58]. In contrast, we observed a difference in the xenon washout parameter of 42% or nearly two standard deviations between AD subjects and healthy controls. Since the xenon washout parameter is a function of cerebral perfusion, our results raise the question of why the xenon washout parameter demonstrates a greater difference between AD participants and healthy controls. While ASL provides a direct measure of cerebral perfusion, our technique incorporates additional factors, such as T1 and the xenon lipid diffusion coefficient, that may be affected in AD patients.

It is possible that the xenon washout parameter is affected by a difference in T1 relaxation time values between healthy controls and AD participants. It is interesting to speculate that small changes in T1 may be caused by the abundance of trace metals in the brain of AD participants [10,80,81,82,83].

An alternative hypothesis is that the partition coefficient of xenon in the brain tissues is different in AD participants compared to healthy controls. It is well established that Aβ affects the membrane properties of the brain [84,85,86,87,88,89]. We speculate that because of the increased membrane permeability in AD brain tissue, the partition coefficient of xenon in the brain of AD participants is increased, creating a reservoir of xenon that causes a slower wash out. Additionally, AD is known to breakdown the blood brain barrier (BBB) [90]. This breakdown may affect the clearance of xenon from the brain tissue into the blood. However, it is more likely that this phenomenon would cause increased xenon clearance (and hence faster washout in AD patients) rather than our observations of greater retained xenon in AD brains. This biomarker may offer some advantages over existing biomarkers, especially those relying on CSF. Our proposed biomarker does not require an invasive spinal tap and, so far in our preliminary results, shows a greater difference between AD participants and healthy controls compared to CSF Aβ values.

Like all techniques, this proposed biomarker has a number of limitations. First, it requires an expensive and highly specialized ^129^Xe polarizer to polarize the ^129^Xe gas. Secondly, this technique relies on the patient holding their breath for 20 s, a task that some individuals with dementia could have difficulty with.

Moreover, this study has notable limitations. Firstly, it relied on a small number of subjects precluding accurate determination of the sensitivity and specificity of this technique. Second, because of the small sample size, we were unable to infer the predictive power of this study or to correlate disease severity with the xenon washout parameter value. Lastly, we were unable to differentiate whether our proposed biomarkers are indicative of just AD or all dementias, or even, any neurologic disease. Regardless of these limitations, the proposed ^129^Xe xenon washout parameter biomarker has the potential for validation with a larger sample size study to determine both its accuracy and predictive power of impending AD. Additionally, future experiments could attempt to correlate the Montreal Cognitive Assessment (MOCA) scores to ^129^Xe retention and the xenon washout parameter; these biomarkers could be tested as a potential correlate to future AD risk.

In conclusion, we demonstrate a difference in the ^129^Xe retention between AD participants and healthy controls. Additionally, we introduce the termed xenon washout parameter which accounts for changes in cerebral perfusion, and differences in ^129^Xe T1 relaxation and lipid partition coefficients associated with AD pathology. The xenon washout parameter is considerably different in healthy controls and AD participants with little overlap between the two groups.

## Figures and Tables

**Table 1:** Statistics of healthy controls & Alzheimer’s disease (AD) Characteristics ± SD.

	Healthy Controls (*n* = 4)	Alzheimer’s Participants (*n* = 4)
Mean Age (years)	70.0 ± 4.5	71.3 ± 6.2
Age of AD Diagnosis	N/A	64.8 ± 4.3
Sex	2 males/2 females	3 males/1 female
MoCA Score	28 ± 1	21 ± 3
Norm. Xe-WM signal @ 60 s	0.200 ± 0.0163	0.458 ± 0.0531
Norm. Xe-GM signal @ 60 s	0.174 ± 0.0252	0.465 ± 0.569
Xe Washout Parameter–WM	0.073 ± 0.021	0.051 ± 0.025
Xe Washout Parameter−GM	0.094 ± 0.021	0.055 ± 0.029

## Supplementary Materials

The following are available online at http://www.mdpi.com/2075-4418/8/2/41/s1.

## Trial Registration

More information regarding this clinical trial can be found at clinicaltrials.gov NCT02638519.
