# Supplementary material for: Inhaled Xenon Washout as a Biomarker of Alzheimer’s Disease
Source: Diagnostics (Basel). 2018 Jun 6;8(2):41. doi: 10.3390/diagnostics8020041 (PMC6023430; doi:10.3390/diagnostics8020041)
Supplement: Supplementary file 1 [file diagnostics-08-00041-s001.pdf]

Inhaled Xenon Washout as a Biomarker of Alzheimer's Disease

Francis T. Hane [a, b], Tao Li [a], Jennifer-Anne Plata [a], Ayman Hassan [c], Karl Granberg [c], Mitchell S. Albert [a, b, d, †]

Supplementary Information

1. A 32x32x3 tensor of the 3 dynamic Xe MR k-space is exported from Philips MR scanner to Matlab for processing.

$$\begin{bmatrix} 3 & 0 & 1 & 0 & 0 & 2 \\ 45 & 21 & 0 & 22 & 35 & 2 \\ 19 & 5 & 0 & 17 & 10 & 5 \\ 17 & 26 & 0 & 0 & 0 & 23 \\ 39 & 21 & 23 & 4 & 0 & 1 \\ 5 & 0 & 2 & 33 & 15 & \dots \end{bmatrix} \begin{bmatrix} 3 & 0 & 2 & 0 & 0 & 2 \\ 12 & 33 & 7 & 0 & 22 & 2 \\ 12 & 4 & 0 & 12 & 8 & 4 \\ 15 & 24 & 3 & 0 & 0 & 20 \\ 33 & 12 & 18 & 4 & 0 & 3 \\ 4 & 3 & 2 & 16 & 12 & \dots \end{bmatrix} \begin{bmatrix} 0 & 2 & 0 & 02 & 0 & 1 \\ 9 & 9 & 3 & 0 & 8 & 2 \\ 7 & 5 & 0 & 8 & 9 & 5 \\ 6 & 5 & 0 & 0 & 2 & 3 \\ 1 & 6 & 7 & 4 & 2 & 1 \\ 5 & 0 & 2 & 6 & 1 & \dots \end{bmatrix}$$

2. The k-space matrices are converted to MR images via a Fast Fourier Transform (FFT) algorithm using a custom Matlab script.

½ way into breath hold

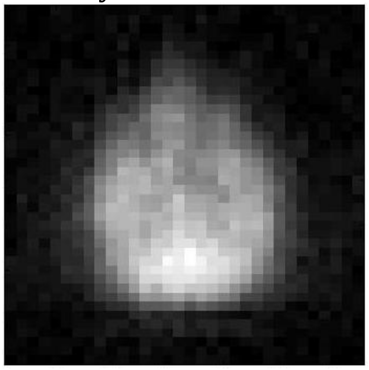

Figure S1

End of breath hold

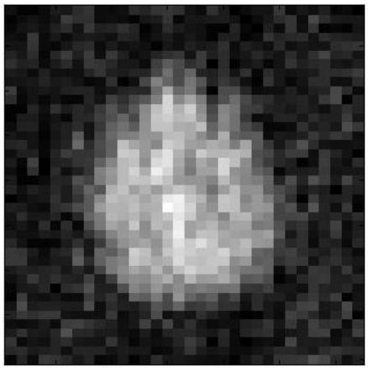

Figure S2

10 sec post breath hold

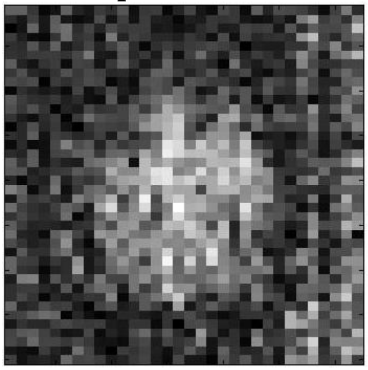

Figure S3

3. The β-parameter of each pixel is calculated by fitting the values of each pixel in each dynamic scan to Equation 2.

Axial

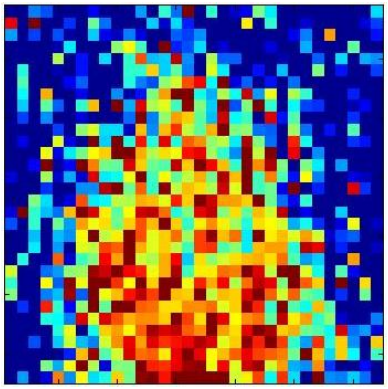

Figure S4

Sagittal

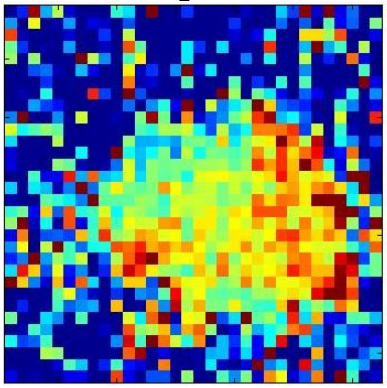

Figure S5

4. β-parameter maps are signal averaged by taking the mean of each pixel for all subjects.

Axial

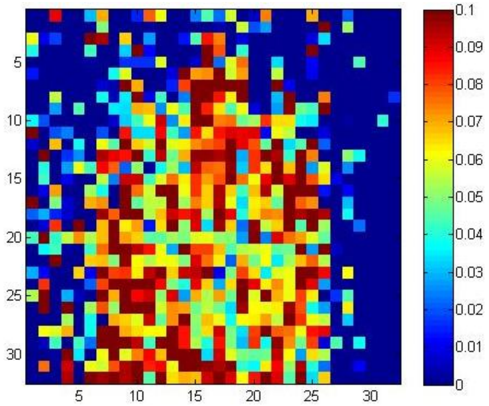

Figure S6

Sagittal

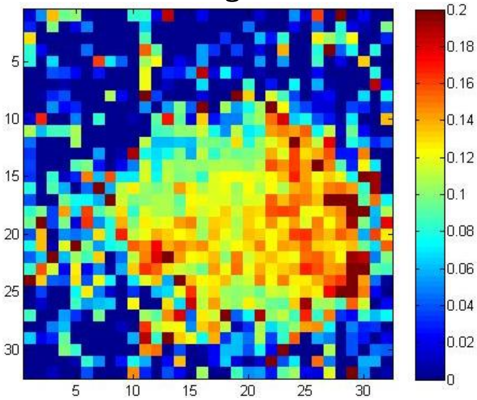

Figure S7

5. A mask is created to remove the pixels outside of the brain region to create an overlaid β-parameter map. A transparency filter is added to the β-parameter map so that the underlying anatomical MRI is visible.

Axial

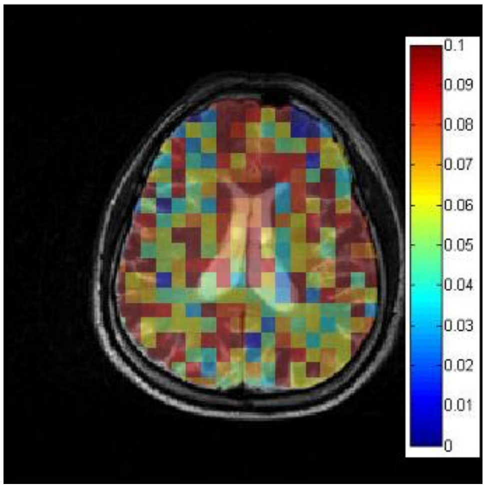

Figure S8

Sagittal

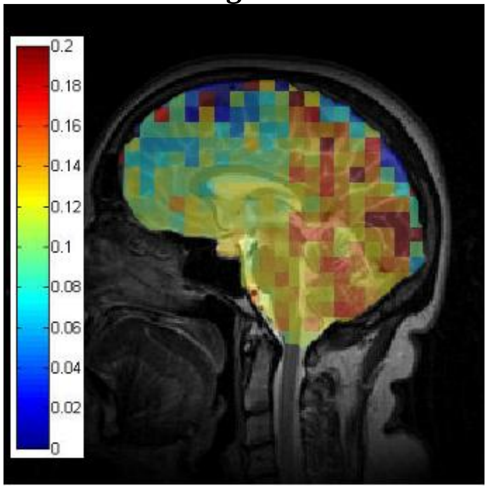

Figure S9

Table S1 - Washout Parameter Map Processing Scheme

Matlab β-Parameter Map Processing Script

```
close all;
clear all;
clc;

%% *Set Reconstruction Parameters*
Recon_size = 32;
NSA = 6;
MeanImage = zeros(Recon_size,Recon_size);

for n = 1:NSA

% *Read in Xe fMRI Data*
    [filename,pathname] = uigetfile('*.list','Select *.list file for
      Stimulation scan');
    s_listfile = [pathname filename];
    [s_data,s_kk,s_kk_s,s_parms] = GetData_listdata(s_listfile);

    %% *Process k-space Data & Reconstruct Images
    data = prcs(s_kk_s,Recon_size);

    %% *Calculate beta*

    for i = 1:Recon_size
        for j = 1:Recon_size
           beta(i,j) = washoutbetai([data(i,j,1) data(i,j,2) data(i,j,3)],0);
        end
    end

    MeanImage = (MeanImage + beta)/n;

end

%% display figure
figure(4)
imagesc(MeanImage)
axis square
colormap jet
colorbar
caxis ([0 0.1])
```

```
function [p_data] = prcs(r_data, zf_size)
data_size = size(r_data);
index = size(data_size);

if index(2) == 3
    for i = 1:data_size(3)
        temp(:,:,i) = zf(r_data(:,:,i), zf_size);
        temp2(:,:,i) = k2i(temp(:,:,i));
        truncpoint = [floor(zf_size/2), ceil(zf_size/2*3)];
        p_data(:,:,i) = trnc(temp2(:,:,i));

    end

    N_sample = p_data(1:5,1:5,1); %can change noise area for bigger images
    N_sample_reshape = reshape(N_sample, 5*5,1);
    Noise = std(N_sample_reshape);

    p_data = p_data/Noise;

elseif index(2) == 2

     temp(:,:) = zf(r_data(:,:),zf_size);
     temp2(:,:) = k2i(temp(:,:));
     truncpoint = [floor(zf_size/2) ceil(zf_size/2*3)];
     p_data(:,:,i) = trnc(temp2(:,:,i));

     N_sample = p_data(1:5,1:5,1);
     N_sample_reshape = reshape(N_sample, 5*5,1);
     Noise = std(N_sample_reshape)

     p_data = p_data/Noise;

end
```

```
function [beta] = washoutbetai(data,c)

[~, position] = max(data); % Get the peak value position.

size_data = size(data,2);

% Fitting.
t = 1:size_data;
fa = 10; % flip angle

if c == 0
    a = polyfit(t,log(data),1);

elseif c == 1

    for i = 1:size_data
        data(i) = data(i)/cosd(fa)^(i-1);
    end

    a = polyfit(t,log(data),1);

end

beta = -a(1);
```
